# Paediatric and adult patients from New Caledonia Island admitted to the ICU for community-acquired Panton-Valentine leucocidin-producing *Staphylococcus aureus* infections

**DOI:** 10.1038/s41598-022-15337-w

**Published:** 2022-06-30

**Authors:** O. Imauven, J. Colot, E. Couadau, P.-H. Moury, A. Preault, F. Vincent, Philippe Montravers

**Affiliations:** 1 Service d’Anesthésie-Réanimation et Médecine Périopératoire Rive Droite, APHP, Hôpital Tenon, DMU DREAM, Sorbonne Université, GRC 29, Paris, France; 2 Microbiology Laboratory, Centre Hospitalier Territorial de Nouvelle-Calédonie, 98 835, Dumbéa-Sur-Mer, New Caledonia; 3 Groupe de Bactériologie Médicale et Environnementale, Institut Pasteur de Nouvelle-Calédonie, 98845 Nouméa Cedex, New Caledonia; 4 Intensive Care Unit, Médipôle Koutio, Centre Hospitalier Territorial de Nouvelle-Calédonie, 98 835, Dumbéa-Sur-Mer, New Caledonia; 5 Unité d’Epidémiologie, Institut Pasteur de Nouvelle-Calédonie, 98845 Nouméa Cedex, New Caledonia; 6 Pôle Anesthésie-Réanimation, CHU Grenoble Alpes, CS 10217, Grenoble Cedex 9, France; 7 HP2 Laboratory, U1042, INSERM, Grenoble Alpes University, CS 10217, Grenoble Cedex 9, France; 8 UMR 1152 PHERE, INSERM, Université de Paris, Paris, France; 9 Département d’Anesthésie-Réanimation, CHU Bichat-Claude Bernard, DMU PARABOL, APHP, 46 Rue Henri Huchard, 75018 Paris, France

**Keywords:** Microbiology, Diseases, Risk factors, Signs and symptoms

## Abstract

Severe infections involving Panton-Valentine leukocidin-producing *Staphylococcus aureus* (PVL + Sa) are increasing. This monocentre, retrospective descriptive cohort assessed clinical characteristics and outcome of paediatric and adult patients admitted for community-acquired PVL + Sa infections to the unique intensive care unit (ICU) on New Caledonia Island. Overall, 72 patients (including 23 children) admitted for acute respiratory failure (42%), sepsis/septic shock (21%), and/or postoperative care (32%) were analysed. Most patients had pulmonary (64%), skin/soft tissue (SSTI) (54%) and/or osteoarticular (38%) infections. Multifocal infections (≥ 2 sites) and bacteraemia were reported in 65% and 76% of the patients, respectively. Methicillin-resistant *S. aureus* isolates were reported in 61% of adult cases versus 30% in children (p < 0.05). Mechanical ventilation, vasoactive support and source control were administered in 53%, 43% and 58% of the patients, respectively. All paediatric patients received adequate empirical antibiotic therapy versus 30/49 adults (p < 0.001). Adequate documented therapy was obtained ≤ 72 h in 70/72 (97%) patients. Death was only reported in adults (n = 10 (14%)), mainly during pulmonary infection (22%), SSTIs (21%) and bacteraemia (24%)). In summary, in ICU patients from New Caledonia Island the clinical presentation of severe community-acquired PVL + Sa infections seems different from Western European observations with high rates of multifocal infections and methicillin-resistant strains.

## Introduction

Over the last decade, the emergence of Panton-Valentine leukocidin (PVL)-producing *Staphylococcus aureus* (PVL + Sa) strains has become an increasing concern due to reports in the community and its capacity to induce extensive tissue necrosis^1,2^. The most frequent clinical presentations of these community-acquired PVL + Sa infections are necrotising pneumonia and skin and soft tissue infections (SSTIs), often in young patients without comorbidities^3^. Severe musculoskeletal and multifocal infections have also been reported, particularly in children^4,5^.

Many issues involving PVL + Sa are debated, including the geographic variability, the prevalence of methicillin-susceptible (MSSA) and methicillin-resistant (MRSA) strains and their susceptibility profile^3^. While the epidemiology in European and North American countries has been extensively documented^3^, the data from other regions are quite limited. Recent reports from the Southwest Pacific region suggest high rates of community-acquired PVL + Sa infections, especially SSTIs^6–10^. However, reports of critical care cases in this region are very rare. The purpose of the current study was to compare the clinical characteristics and morbidity and mortality rates related to PVL + Sa infections of paediatric and adult ICU patients admitted to a single centre on New Caledonia Island in the Southwest Pacific region.

## Methods

### Study population

This retrospective, monocentre, noninterventional cohort study was performed at the Regional General Hospital of Noumea, New Caledonia, a French special territory in the southwest Pacific Ocean. This 528-bed hospital (CHT de Nouméa) with a 20-bed ICU and 12-bed intermediate care unit is the only emergency facility for a community of 270,000 inhabitants, including those living on small surrounding islands. The study was performed according to the relevant guidelines and regulations of French Law. The protocol and all experimental methods were approved by the Local Institutional Review Board (Comité d’Éthique, Centre Hospitalier Territorial Gaston-Bourret de Nouvelle Caledonie 2020-004; 2020/08/31) which waived the need for signed informed consent due to the observational retrospective nature of the study, but parental agreement was obtained for the use of observations in children.

All consecutive paediatric and adult cases of proven community-acquired PVL + Sa infections admitted to the ICU over a 10-year period (March 2010 to September 2020) were retrospectively included in the study. The identification of the cases and selection of participants with a diagnosis of PVL + Sa infection were made using the database of the health information system of the hospital. Patients not admitted to the ICU and those treated for health care-associated *S. aureus* infections were excluded.

### Patient characteristics

All data were obtained through archived and computerised patient records and were collected in adherence with Strengthening the Reporting of Observational Studies in Epidemiology (STROBE) guidelines^11^. Demographic data, past medical history and underlying diseases, clinical features, and radiological and biological characteristics were collected upon ICU admission. A specific focus was made on multifocal tissue and organ infections involving two or more sites of PVL + Sa infections (bacteraemia excluded from count). The severity score was assessed using the SAPS-II score on the day of ICU admission^12^. Therapeutic management, organ-supportive therapy, procedures used for source control, and anti-infective therapies were assessed.

### Definitions

PVL + Sa infections were defined as a positive culture from a normally sterile site obtained at the hospital or as ICU admission. The definitions used for the site of infection were those recommended by the guidelines of the French Society of Anaesthesiology and Critical Care Medicine^13^. Sepsis and septic shock were classified according to the Third International Consensus Definition on Sepsis and Septic Shock^14^. Acute respiratory distress syndrome (ARDS) was classified according to international definitions^15^. In line with previous publications, severity criteria for PVL + Sa infections were defined as fever > 39 °C, haemoptysis and leucopenia < 3000/mm^16,17^. Morbidity criteria were defined as severe complications reported during the ICU stay (e.g., ARDS, multiple organ failure, thromboembolic episodes).

### Microbiological data

Microbiological samples, including blood cultures, were collected at the time of ICU admission and repeated in clinical situations with suspected sepsis or bacteraemia. Samples were collected during source control procedures. Swabs and samples from nonsterile sites were not considered.

The clinical samples were collected and analysed according to the recommendations of the Antibiogram Committee of the French Society for Microbiology^18^. All morphologically distinct colonies were identified by standard bacteriologic techniques and tested for antibiotic susceptibility by the disc diffusion method according to French recommendations^19^. Methicillin-resistant *S. aureus* (MRSA) strains were noted. Until 2014, the diagnosis of PVL + Sa infection was based on microbiological results from the French Reference Centre for Staphylococci. Since 2014, a multiplex PCR technique developed locally has been used for routine identification of the LukS-PV gene.

The detection of PVL in the *S. aureus* genome was carried out using real-time PCR based on SYBR-Green I technology targeting the LukS-PV gene. In parallel, a PCR targeting the gyrase gene confirmed the identification of the *S. aureus* species and played the role of internal control. To destroy the cell wall of *S. aureus*, 300 μL of bacterial suspension (5 UFC in 1 mL of 1 × PBS buffer) were brought into contact with 200 μL of lysozyme solution at 50 mg/mL (Roche Diagnostics, Meylan, France), then incubated for 30 min at 37 °C. Then, 200 μL of this suspension were extracted using the MagNA Pure LC2.0 (Roche Diagnostics) or the QIA Symphony (Qiagen, Hilden, Germany) with an elution volume of 50 μl.

PCR amplification was performed using the LightCycler® FastStart DNA Master SYBR Green I (Roche Diagnostic) kit on a 20 μL total volume containing 2 μL of diluted extracted strain DNA (50 μL extracted DNA + 650 μL H2O PCR grade nuclease free) and 18 μL of PCR mix containing 0.3 μMol of the PVL-specific primers LukS 865 (Pvl LukS1): 5′CAAATGCGTTGTGTATTCTAGATCCT3′ and LukS 765 (Pvl LukS2): 5′AATAACGTATGGCAGAAATATGGATGT3′^20^.

Cycling conditions were 95 ˚C for 10 min, followed by 30 cycles of 95 ˚C for 10 s and 58 ˚C for 30 s with data collection at the end of each extension cycle. Finally, a melting curve analysis was done with continuous fluorescence acquisition during temperature increase from 60 °C to 97 °C, at 0.05 °C/s. The positive control was a *S. aureus* PVL positive strain kindly provided by the French Reference Centre for Staphylococci and confirmed by sequencing. The negative control was PCR-grade ultrapure water.

### Outcome

The patients were followed until their ICU discharge, transfer to another facility or death. No postmortem examination was performed. In line with our objectives, the primary study endpoints were all-cause mortality rate at ICU discharge and risk factors for death. Secondary endpoints were to compare the paediatric and adult outcomes and to assess severe complications related to PVL + Sa infection observed during the ICU stay.

### Statistical analysis

The median and interquartile range (IQR) were calculated for continuous variables, and numbers and proportions were calculated for categorical parameters. Missing data were not replaced in the final dataset. Comparisons between groups were performed using Fisher’s exact test or the chi-square test for categorical variables, and continuous variables were compared using the Wilcoxon test. The study population was split into three periods (2010–14, 2015–2017 and 2018–2020) to assess temporal changes, and comparisons of relevant clinical features were performed using the chi-square test and Kruskal–Wallis test. The predictive factors of death were assessed with univariate analysis, as the low number of events limited the value of a multivariate analysis.

The results are presented as odds ratios (ORs) with their 95% confidence intervals (CIs) for univariate analyses. As the study was strictly retrospective and observational, no power calculation was performed^21^. The statistical analyses were performed using JMP software (SAS Institute Inc., Cary, NC, USA). The significance threshold was a priori set at a significance of 0.05.

## Results

### Study population

During the 10-year study period, a total of 14,197 *S. aureus* cultures were recorded from the CHT de Nouméa, including 3,674 (20.7%) methicillin resistant isolates. Among the 778 patients admitted to the ICU for *S. aureus* infections (including 199 (25.6%) MRSA infections), 72 (9%) critically ill patients were managed for severe community-acquired PVL + Sa infection. The study population is presented in Table 1. No significant change over time was observed among the studied parameters (supplementary material Tables S1 and S2).

**Table 1:** Clinical characteristics at the time of ICU admission.

Clinical characteristics	Missing data	Paediatric population (n = 23)	Adult population (n = 49)	P value
Male gender, n (%)	0	15 (65)	34 (69)	NS
Age, years, median [IQR]	0	7 [1–12]	54 [34–64]	< 0.001
**Comorbidities and underlying diseases**	0	3 (13)	32 (65)	< 0.001
Diabetes mellitus, n (%)	0	–	12 (24)	–
Tobacco smoking, n (%)	0	–	17 (35)	–
Alcohol consumption (> 40 g/day), n (%)	0	–	9 (18)	–
Obesity, n (%)	0	–	5 (10)	–
Obstructive sleep apnoea, n (%)	0	–	5 (10)	–
Hypertension, n (%)	0	–	13 (27)	–
Chronic respiratory disease, n (%)	0	3 (13)	11 (22)	NS
Chronic renal insufficiency, n (%)	0	–	2 (4)	–
Peripheral vascular arterial disease, n (%)	0	–	2 (4)	–
Steroid therapy over the last 3 months, n (%)	0	–	3 (6)	–
Other immunosuppressive therapy, n (%)	0	–	4 (8)	–
Furunculosis, n (%)	6/5	3 (13)	15 (30)	NS
Delay between the first symptoms and ICU admission < 48 h, n (%)	2/5	5 (24)	11 (22)	NS
Cutaneous injury in the last 30 days, n (%)	2/2	11 (48)	30 (61)	NS
Nonsteroid anti-inflammatory therapy in the last 7 days, n (%)	0	1 (4)	4 (8)	NS
Influenza-like infection prior to hospitalisation, n (%)	1/0	5 (22)	14 (29)	NS
**Main causes for ICU admission**				
Acute respiratory failure, n (%)	0	8 (35)	22 (45)	NS
Sepsis/Septic shock, n (%)	0	3 (13)	12 (24)	NS
Postoperative management, n (%)	0	11 (48)	12 (24)	NS
Miscellaneous, n (%)	0	1 (4)	3 (6)	NS
**Severity criteria**	0			
Fever > 39 °C, n (%)	0/2	15 (65)	22 (45)	NS
Haemoptysis, n (%)	1/1	1 (4)	4 (8)	NS
Leucopenia (< 3000/mm^3^), n (%)	7/14	2 (9)	4 (8)	NS
Thrombocytopenia (< 150,000/mm^3^), n (%)	8/16	10 (43)	19 (39)	NS
SAPS II score, median [IQR]	0	20 [14–28]	34 [26–57]	< 0.001
**Radiography and/or pulmonary CT scan**	0			
Unilobar infiltrates, n (%)	0	1 (4)	3 (6)	NS
Multilobar infiltrates, n (%)	0	20 (87)	36 (73)	NS
Excavated nodules/pulmonary abscesses/necrotizing pneumonia, n (%)	0	12 (52)	18 (37)	NS
Pleural effusion, n (%)	0	10 (43)	19 (39)	NS
Methicillin-resistant *Staphylococcus aureus*, n (%)	0	7 (30)	30 (61)	< 0.05
Bacteraemia, n (%)	0	19 (83)	36 (73)	NS
Multifocal infection, n (%)	0	16 (70)	31 (63)	NS
Pulmonary infection, n (%)	0	14 (61)	32 (65)	NS
Skin and soft tissue infection, n (%)	0	8 (35)	31 (63)	< 0.05
Osteoarticular, n (%)	0	13 (57)	14 (29)	< 0.05
Muscles, n (%)	0	5 (22)	5 (10)	NS
Kidney and urinary tract, n (%)	0	1 (4)	6 (12)	NS
Endocarditis, n (%)	0	1 (4)	5 (10)	NS
Epidural, n (%)	0	1 (4)	3 (6)	NS
Brain, n (%)	0	–	2 (4)	–
Liver, n (%)	0	–	2 (4)	–
Eye, n (%)	0	–	1 (2)	–
Pericarditis, n (%)	0	3 (13)		
Parotid gland, n (%)	0	1 (4)	–	–
Sinusitis, n (%)	0	1 (4)	–	–

### Clinical presentation at ICU admission

A predominance of male patients (49/72, 68%) was observed. Comorbidities were mainly reported in adult patients: 32/49 (65%) adults versus 3/23 (13%) children (asthma in all three); p < 0.01 (Table 1).

The main reasons for ICU admission were acute respiratory failure (30/72 (42%)), sepsis/septic shock (15/72 (21%)), and postoperative care following surgical debridement (23/72 (32%)). Most patients (58/72 (81%)) were admitted to the ICU with symptoms that developed after ≥ 48 h (median [IQR] delay: 4 [3;7] days). The severity criteria did not differ between adult and paediatric patients. However, the SAPS-II score measured at 31 [16; 53] on the day of ICU admission was significantly higher in adult cases (Table 1).

Most patients had pulmonary (46/72 (64%)), SSTI (39/72 (54%) and osteoarticular (27/72 (38%)) infections. SSTIs were predominantly observed in adults, while osteoarticular infections were more frequently observed in children (Table 1). A single-site infection was observed in 18 adult patients and 7 children: pleuropulmonary infection (n = 15, including 3 children), SSTI (n = 7, one child), osteoarticular infections (n = 3, 2 children) and one paravesical abscess in a child. A predominance of multifocal infections was reported in 47/72 (65%) patients: 2 sites n = 23 including 6 children, 3 sites n = 19 (8 children), and 4 sites n = 5 (2 children). In patients with multifocal infection, we observed a predominance of male patients (37/47 (79%)) versus 12/25 (48%) male patients with a single site of infection (p < 0.05). The same trend was observed in paediatric cases: 12/23 (52%) male children vs. 3/23 (13%) male children with a single location (p < 0.05).

Most cases had bacteraemia (55/72 (76%) patients). Bacteraemia was observed in 41/47 (87%) patients (including 14 children) with multifocal infections versus 14/25 (56%) patients with a single-site infection (p = 0.0502).

Overall, MRSA isolates were reported in 37/72 (51%) cases with a predominance in adult patients (Table 1). All MRSA isolates were susceptible to vancomycin, and 95% of the isolates were susceptible to clindamycin. A lower proportion of MRSA was cultured in patients with multifocal infections: 20/47 (43%) patients versus 17/25 (69%) patients with a single-site infection (p < 0.05).

### Pleuropulmonary infections

Among the 46/72 (64%) patients admitted for pleuropulmonary infection, a multifocal infection was reported in 31/46 (67%) cases, including 11/14 (79%) children. Bacteraemia was associated in 37/46 (80%) cases, including 11/14 (79%) children. The median SAPS-II score on ICU admission was 31 [18; 56], with an increased severity in adult patients (44 [23; 58] versus 21 [15; 29] in paediatric patients; p < 0.01). A flu-like syndrome was reported in 16/46 (35%) patients. A nasal swab performed for polymerase chain reaction diagnosis for influenza viruses in 42/72 (58%) patients (including 12/14 (86%) children) led to eight flu-positive cases, including one child. Haemoptysis was reported in five patients before ICU admission and in nine patients (3 children and 6 adults) during the ICU stay. Acute respiratory failure was reported in 26/46 (57%) patients, leading to mechanical ventilation in 27/46 (59%) patients (5/14 (36%) children). In addition, vasoactive support was used in 22/46 (48%) cases (3/14 (21%) children). Chest CT scans identified images disseminated in several pulmonary lobes in 43/46 (93%) cases (13/14 (93%) children), including pulmonary abscesses in 24/46 (52%) patients (7/14 (50%) children) and pleural effusion in 23/46 (50%) patients (8/14 (57%) children).

Pleural and pulmonary samples were obtained in 41/46 (89%) patients (11/14 (79%) children) and led to 21/41 (51%) positive microbiological PVL + Sa cultures (3 children); these included bronchoalveolar lavage (n = 8), endotracheal aspirates (n = 7, of which 2 were children), sputum (n = 5), and pleural fluid (n = 1 in a child). Positive blood cultures were reported in 21/25 patients without positive pulmonary samples (including 8 children). Overall, MRSA strains were identified in 24/46 (52%) patients (6 children), including 20/46 (43%) bacteraemic patients (5 children).

### Skin and soft tissue infections

Among the 39/72 (54%) patients with SSTIs (mainly limb (n = 20, including 8 children), buttock (n = 10, 1 child), and head and neck (n = 8) locations), 16/39 (41%) patients including two children had a previous history of furunculosis. A cutaneous injury (mainly furuncles) was reported in the month preceding admission in 34/39 (87%) cases, including seven children. Nonpurulent infections (necrotising infections and cellulitis) were observed in 11 cases (1 child), and purulent infections (furuncles and abscesses) were reported in 26 cases (6 children). The median SAPS-II score on ICU admission was 31 [16; 53], with an increased severity in adult patients (34 [19; 55] versus 16 [11; 27] in paediatric patients; p < 0.05). Multifocal infections were reported in 32/39 (82%) patients, including 7/8 (88%) children. Bacteraemia was associated in 32/39 (82%) cases, including 7/8 (88%) children.

Positive microbiological PVL + Sa cultures were obtained from abscess drainages and surgical debridements (17/39 (43%) patients, including 3 children). Methicillin-resistant PVL + Sa strains were identified in 22/39 (56%) patients (including 2/8 (25%) children) and included 18 bacteraemic patients (including 2/7 (29%) children).

### Other sites of infections

Osteoarticular infections were reported in 27/72 (38%) patients, including 13/23 (57%) children: nine osteomyelitis and seven spondylodiscitis (8 and 2 children, respectively). Four epidural infections were reported, one in a child. The other most frequent sites of infection were muscles (n = 10 patients including 4 children), involving psoas (n = 6, 2 children), multiple muscular abscesses (n = 2) and pyomyositis (n = 4, 2 children), urinary tract (n = 6, 1 child) including kidney abscesses (n = 5) and prostatitis (n = 1), and endocarditis (n = 6, 1 child). The other sites of infection are reported in Table 1.

### Initial therapeutic management

The main organ supportive therapies are summarised in Table 2. Mechanical ventilation and vasoactive support were administered in 38/72 (53%) and 31/72 (43%) patients, respectively, with a predominance in adult patients. Surgical source control and debridement were performed in 42/72 (58%) cases: abscess, cellulitis and/or myositis debridement and drainage in 31 patients, including eight children; spinal, articular and/or bone debridement and drainage in ten patients, including seven children; thoracic and pleural drainage in four patients, including one child; pericardial drainage in three children; and removal of foreign material in two patients. Extracorporeal membrane oxygenation was used in two children.

**Table 2:** ICU management and outcome.

Clinical characteristics	Missing data	Paediatric population (n = 23)	Adult population (n = 49)	P value
**Organ support**				
Mechanical ventilation	0	7 (30)	31 (63)	< 0.05
Vasoactive support, n (%)	0	5 (22)	26 (53)	< 0.05
Extracorporeal membrane oxygenation, n (%)	0	2 (9)	–	–
**Source control**				
Surgical procedure for source control, n (%)	0	17 (74)	25 (51)	NS
Pleural drainage, n (%)	0	5 (22)	7 (14)	NS
**Empirical antibiotic therapy**				
Combination therapy, n (%)	0			
**Adequate empirical antibiotic therapy, n (%)**		22 (96)	40 (82)	NS
Aminoglycosides, n (%)		23 (100)	30 (61)	< 0.001
Third-generation cephalosporin, n (%)		21 (91)	23 (47)	< 0.001
Clindamycin, n (%)		13 (57)	19 (39)	NS
Piperacillin/tazobactam, n (%)		17 (74)	14 (29)	< 0.001
Vancomycin, n (%)	0	–	12 (24)	–
Macrolides, n (%)		3 (13)	11 (22)	NS
Documented antibiotic therapy		1 (4)	9 (18)	NS
Combination therapy, n (%)		23 (100)	39 (80)	NS
**Adequate documented antibiotic therapy within 72 h, n (%)**		23 (100)	47 (96)	NS
Aminoglycosides, n (%)		15 (65)	15 (31)	< 0.01
Clindamycin, n (%)		18 (78)	31 (63)	NS
Piperacillin/tazobactam, n (%)		–	8 (16)	–
Vancomycin, n (%)		4 (17)	21 (43)	< 0.05
Linezolid, n (%)		3 (13)	9 (18)	NS
Oxacillin, (%)		8 (35)	8 (16)	NS
Intravenous immunoglobulin therapy, n (%)		7 (30)	12 (24)	NS
**Main complications**	0			
Acute respiratory distress syndrome, n (%)	0	4 (17)	18 (37)	NS
Deep venous thrombosis/pulmonary embolism, n (%)	2	4 (17)	10 (20)	NS
Multiple organ failure, n (%)	0	2 (9)	17 (35)	< 0.05
Duration of mechanical ventilation of survivors, median days [IQR]	0	0 [0–2]	11 [2–21]	< 0.01
Duration of ICU stay of survivors, median days [IQR]	0	7 [5–12]	8 [3–26]	NS
Death, n (%)	0	–	10 (20)	–

The most frequently prescribed antibiotic agents are presented in Table 2. Adequate antibiotic therapy was administered in 18 patients (including 11 children) before ICU admission, all for MSSA infection. All patients received empirical antibiotic therapy after microbiological samples. Adequate empirical antibiotic therapy was given in 53/72 (74%) of the patients within the first 24 h after admission. All paediatric patients received adequate empirical therapy versus 30/49 adults (p < 0.001). Overall, empirical therapy was active against 34/35 (97%) MSSA and 19/37 (51%) MRSA strains. A significantly increased proportion of adequate empirical antibiotic therapy was observed in patients with multifocal infections: 40/47 (85%) versus 13/25 (52%) patients with a single-site infection (p < 0.01). Adequate documented therapy was given within the first 72 h after admission in 70/72 (97%) patients (1 methicillin-susceptible *S. aureus* and 1 MRSA strain not adequately targeted in adult patients), including 62/72 (86%) patients receiving combination therapy. Antibiotics with antitoxin properties (linezolid or clindamycin) were introduced within the first 72 h after admission in 58/72 (81%) patients, including 20/23 (87%) children.

During the ICU stay, intravenous immunoglobulins were given in 19/72 (26%) patients (including 7 children), with severe multifocal (n = 17 including 6 children), bacteraemic (n = 18 including 6 children) infections [SSTI n = 14 (3 children), pneumonia n = 13 (3 children)], including 8 cases (3 children) in the first 24 h after ICU admission. All these patients received combination therapy using clindamycin (n = 17) and/or linezolid (n = 3).

### Outcome

Overall, three patients (one adult with endocarditis requiring emergent surgery and two children with severe ARDS receiving ECMO) were transferred to Australia with a median delay of 6 [2; 7] days. Death was reported in 10/72 (14%) patients. All the nonsurvivors were adult patients, including 7/32 (22%) patients with pulmonary infection, 6/29 (21%) with SSTIs, and 8/33 (24%) bacteraemic patients with a median delay of 8 [2; 22] days. No additional death was reported after ICU discharge. Death was significantly associated with a high SAPS-II score (79 [53; 89] vs. 28 [16; 45] in survivors; p < 0.001), the presence of septic shock (p < 0.001), and orotracheal intubation (p < 0.01) upon ICU admission (Table 3). A trend towards a decreased mortality rate in adult patients was observed over time: 6/20 (30%) deaths in the period from 2010 to 14 versus 2/23 (9%) in 2015–17 and 2/26 (8%) in 2018–20 (p = 0.08) (Table S2).

**Table 3:** Comparison of clinical characteristics and management of survivors and deaths in adult patients in univariate analysis.

Clinical characteristics	Survivors	Deaths	Univariate analysis	
	(n = 39)	(n = 10)	OR [95%CI]	p value
Male gender, n (%)	28 (72)	6 (60)	0.58 [0.13–2.5]	0.47
Age, years, median [IQR]	54 [34–61]	61 [47–73]	2.15 [0.52–8.92]	0.31
Diabetes mellitus, n (%)	8 (21)	4 (40)	2.58 [0.58–11.49]	0.23
Obesity, n (%)	3 (8)	2 (20)	3.03 [0.42–21.27]	0.27
Hypertension, n (%)	8 (21)	5 (50)	3.87 [0.89–16.75]	0.10
Chronic respiratory disease, n (%)	10 (26)	1 (10)	0.32 [0.03–2.87]	0.42
Furunculosis, n (%)	11 (28)	4 (40)	1.72 [0.40–7.24]	0.47
Delay between the first symptoms and ICU admission < 48 h, n (%)	8 (21)	3 (30)	1.66 [0.35–7.90]	0.67
Cutaneous injury in the last 30 days, n (%)	25 (64)	5 (50)	0.56 [0.14–2.27]	0.48
Influenza-like infection prior to hospitalisation, n (%)	10 (29)	4 (40)	1.93 [0.45–8.33]	0.44
**Cause for ICU admission**				
Acute respiratory failure, n (%)	18 (46)	4 (40)	0.77 [0.19–3.19]	1.00
Sepsis/Septic shock, n (%)	7 (18)	5 (50)	4.57 [1.03–20.18]	0.05
**Severity criteria**				
Fever > 39 °C, n (%)	17 (44)	5 (50)	1.29 [0.32–5.20]	0.73
Haemoptysis, n (%)	3 (8)	1 (10)	1.33 [0.12–14.38]	1.00
Leucopenia (< 3000/mm^3^), n (%)	3 (8)	1 (10)	1.33 [0.12–14.38]	1.00
SAPS II score, median [IQR]	133 [19–53]	79 [53–89]	1.44 [1.65–125.4]	< 0.01
Bacteraemia, n (%)	28 (72)	8 (80)	1.57 [0.29–8.59]	0.71
Methicillin-resistant *Staphylococcus aureus*	22 (56)	8 (80)	3.09 [0.58–16.50]	0.28
Multifocal infection, n (%)	26 (67)	5 (50)	0.50 [0.12–2.04]	0.46
Pulmonary site of infection, n (%)	25 (64)	7 (70)	1.31 [0.29–5.87]	1.00
SSTIs, n (%)	25 (64)	6 (60)	0.84 [0.20–3.49]	1.00
**Medical management**				
Mechanical ventilation, n (%)	21 (54)	10 (100)	18.1 [0.98–329]	< 0.01
Vasoactive support, n (%)	16 (41)	10 (100)	29.9 [1.63–546]	< 0.001
Surgical procedure for source control, n (%)	21 (54)	4 (40)	0.57 [0.13–2.34]	0.49
Adequate empiric antibiotic therapy, n (%)	25 (64)	5 (50)	0.56 [0.14–2.27]	0.48
Adequate documented antibiotic therapy within 72 h, n (%)	39 (100)	8 (80)	0.04 [0.002–98]	0.04
Intravenous immunoglobulin therapy, n (%)	8 (21)	4 (40)	2.58 [0.58–11.40]	0.23

The ICU stay was marked by multiple organ failure observed in 19/72 (26%) patients, including two children, leading to ten deaths. Acute respiratory distress syndrome was reported in 22/72 (31%) patients, including four children, and resulted in six deaths. Five of the adult patients and one child who developed ARDS without other organ failure survived. Thromboembolic episodes were reported in 14/72 (19%) cases, including four children (11 patients with multifocal infections, ten patients with SSTI locations, all bacteraemic patients). Chest drainage was performed in 12/72 (17%) patients, including five children, all cases but one admitted for multiple pulmonary infections and/or necrotising pneumonia. A tracheotomy was performed in 3/72 (4%) adult patients for prolonged mechanical ventilation and difficult weaning.

## Discussion

Our data confirm that PVL + Sa strains are responsible for severe infections leading to ICU admission for septic shock, acute respiratory failure and/or emergency drainage and source control. Clinical presentation can be extremely heterogeneous, with a predominance of SSTIs and necrotising pneumonias. A large proportion of patients had multifocal infections involving the lungs, SSTIs, deep space abscesses and/or osteoarticular tissue. The clinical presentation of paediatric cases compared to adult patients was marked by the absence of comorbidities, a slightly lower severity, and similar proportions of pulmonary infections but higher rates of osteoarticular infections. A predominance of MRSA was noted in adult cases and lower proportions in paediatric cases. Empirical antibiotic therapy was suboptimal in adults, while paediatric cases were adequately treated from the early phase of treatment. Adequate documented therapy was quickly achieved in almost all cases.

The infections reported in the current cohort, although with a higher prevalence than in Western countries, remain rare in the ICU setting^3^. The severity of the cases reported in the current cohort stands in the same range as the previously published observations, especially in cases of PVL + Sa pulmonary infections^3,22^. In the patients treated for pulmonary infections, high morbidity and mortality rates were observed for single- and multifocal-site infections. The severity of the disease in our study population might be related to delayed admission after the onset of the disease.

The literature has mentioned several case reports and short series of multifocal PVL + Sa infections in children^4,5,23–27^. However, publications mentioning large cohorts of critically ill children are rare^25,28^. The predominance of pulmonary and osteoarticular PVL + Sa infections has been previously reported in the paediatric literature^17,29^, but our population was almost free of comorbidities compared to previous publications^28^.

In contrast, the data for ICU adult patients are poor. Vandroux et al. reported one case of MSSA myelitis associated with epidural abscesses among three cases of PVL + Sa pneumonia in adult patients on Reunion Island (a French Island in the Indian Ocean)^30^. However, these multifocal features were not confirmed in a larger cohort of 16 patients with PVL + Sa pneumonia from the same location^31^. Few case reports have mentioned multiple-site infections or unconventional septic sites, including prostatic or renal abscesses or mediastinitis^32–34^.

The unconventional presentation with a high rate of multifocal infections observed in both populations is difficult to explain. Frequently delayed admissions, already mentioned above, could explain some of these observations. We cannot exclude the role of bacteraemia (observed in 76% of the cases) in facilitating the dissemination of staphylococci infections. Local spread from one contaminated tissue or organ to another is also a possible mechanism of dissemination, as PVL + Sa carries many virulence factors^35^.

In comparison with metropolitan French observations of PVL + Sa infections, a high rate of MRSA infections was observed in both paediatric and adult populations in our study^17,25,28^. This feature is not a surprise in light of recent reports of staphylococcal infections among South Pacific neighbours of New Caledonia, where high rates of MRSA strains and PVL + Sa infections have been observed in Australia and Fiji^7–10^. In 2002, a first observational study from New Caledonia reported 68 patients with *S. aureus* SSTIs and only 2 MRSA isolates^6^. Interestingly, PVL genes were detected in 48/54 (89%) bacteria, none of which was methicillin resistant. These changes illustrate the growing source of concern related to the development of MRSA clusters. However, the methicillin susceptibility profile does not seem to be a risk factor for multifocal infections. In addition, we did not find any association between methicillin resistance and the severity or prognosis of our patients. However, this point is a debated topic with contradictory results in two French prospective observational cohorts of patients with PVL + Sa community-acquired pneumonia^17^.

The high rate of inadequate empirical antibiotic therapy in adult patients could be explained by the unconventional presentation of many cases and the high incidence of MRSA infections among single-site infections. These mistakes have been corrected accordingly at the documented phase of therapy with a large use of vancomycin. The high prevalence of PVL + Sa and the high prevalence of clindamycin-susceptible MRSA strains among the New Caledonian isolates led the prescribers to rapidly use an antitoxin antibiotic, especially in cases of pulmonary infections. Immunoglobulins were only administered to a few patients, mainly for bacteraemic multifocal infections. No conclusion can be drawn on the use of these specific therapies for PVL + Sa infections because of the heterogeneity of the cohort and the absence of a locally standardised protocol.

Patients with PVL + Sa pulmonary infections have a severe prognosis (20% mortality rate in adult patients), as previously reported. In a recent French cohort, the authors reported a mortality rate of 47% for the ICU stay with a high initial severity^17^. To the best of our knowledge, the mortality rate of PVL + Sa SSTIs has never been reported in adult ICU patients. The absence of death in the paediatric cohort is another point worth mentioning, possibly related to the absence of severe comorbidities in the children and adequate empirical antibiotic therapy. Our data suggest that mortality rates were quite similar between pulmonary and SSTI infections. Moreover, the usual mortality risk factors of age, septic shock, mechanical ventilation requirement, and multiple organ failure were observed in the univariate analysis.

Several limitations need to be emphasised. This study is a retrospective, descriptive, monocentre analysis performed over a prolonged period. However, because of the insular nature of the study centre, the data presented here correspond to an accurate evaluation of severe PVL + Sa infections. The long duration of the investigation questions the changes in standard of care during the study. Although this assumption cannot be ruled out, we did not observe any significant difference related to the prolonged observational analysis. The small number of cases is another limitation. However, the purpose of the current study was purely exploratory. Considering the usual number of cases in previously published papers, our cohort represents a large but heterogeneous panel. Our mortality rates are in the low range compared to previous cohorts, especially for pulmonary infections, which raises the issue of the severity of the cases. The heterogeneity of the cases could explain these observations. However, morbidity features demonstrate the difficult management of these patients.

In summary, we report high proportions of multifocal infections and MRSA strains in a cohort of paediatric and adult ICU patients admitted for severe PVL + Sa infections. The combination of these features has not been reported in the literature. Further investigations are required, including epidemiological analyses at the South Pacific level. Currently, ICU physicians in New Caledonia, aware of the severity of these PVL + Sa infections, include the risk of methicillin resistance in their empirical therapy as soon as PVL + Sa is suspected for pulmonary and SSTI presentations.

## Supplementary Information

Supplementary Tables.

## Data Availability

The datasets generated during and/or analysed during the current study are publicly available from the corresponding author on reasonable request.
