# Supplementary material for: Paediatric and adult patients from New Caledonia Island admitted to the ICU for community-acquired Panton-Valentine leucocidin-producing Staphylococcus aureus infections
Source: Sci Rep. 2022 Jun 30;12:11024. doi: 10.1038/s41598-022-15337-w (PMC9247012; doi:10.1038/s41598-022-15337-w)
Supplement: Supplementary file 1 — Supplementary Tables. [file 41598_2022_15337_MOESM1_ESM.docx]

Supplementary material Table S1**.** Clinical characteristics across three study periods in paediatric patients

| Clinical characteristics | 2010-14 | 2015-17 | | 2018-20 | P value |
| --- | --- | --- | --- | --- | --- |
|  | (n=8) | (n=7) | (n=8) | |  |
| Male gender, n (%)  Age, years, median [IQR] | 5 (63)  9 [4-14] | 6 (86)  7 [1-9] | 4 (50)  5 [1-13] | | NS  NS |
| Furunculosis, n (%) | 1 (13) | 1 (14) | 0 | | NS |
| Delay between the first symptoms and ICU admission <48 hours, n (%)  Influenza-like infection prior to hospitalisation, n (%) | 1 (13)  0 | 3 (43)  2 (29) | 1 (13)  3 (38) | | NS  NS |
| Multifocal infection, n (%)  Bacteraemia, n (%)  Methicillin-resistant Staphylococcus aureus, n (%) | 5 (63)  7 (88)  1 (13) | 6 (86)  5 (71)  2 (29) | | 5 (63)  7 (88)  4 (50) | NS  NS  NS |
| SAPS II score, median [IQR] | 19 [11-32] | 18 [16-32] | | 21 [14-27] | NS |
| Mechanical ventilation  Vasoactive support, n (%)  Surgical procedure for source control, n (%)  Adequate empiric antibiotic therapy, n (%)  Adequate documented antibiotic therapy, n (%)  Intravenous immunoglobulin therapy, n (%) | 3 (38)  2 (25)  5 (63)  8 (100)  8 (100)  3 (38) | 2 (29)  2 (29)  7 (100)  7 (100)  7 (100)  3 (43) | | 2 (25)  1 (13)  5 (63)  8 (100)  8 (100)  1 (13) | NS  NS  NS  NS  NS  NS |
| Main complications  Adult respiratory distress syndrome, n (%)  Multiple organ failure, n (%)  Duration of ICU stay, median days [IQR] | 2 (25)  1 (13)  6 [4-11] | 1 (14)  1 (14)  11 [6-16] | | 1 (13)  0  8 [6-23] | NS  NS  NS |

Supplementary material Table S2**.** Clinical characteristics across three study periods in adult patients

| Clinical characteristics | 2010-14 | 2015-17 | | 2018-20 | P value |
| --- | --- | --- | --- | --- | --- |
|  | (n=15) | (n=16) | (n=18) | |  |
| Male gender, n (%)  Age, years, median [IQR] | 11 (73)  56 [32-62] | 10 (63)  43 [31-59] | 13 (72)  55 [48-65] | | NS  NS |
| Absence of comorbidities  Furunculosis, n (%) | 7 (47)  5 (33) | 6 (38)  6 (38) | 4 (22)  4 (22) | | NS  NS |
| Delay between the first symptoms and ICU admission <48 hours, n (%)  Influenza-like infection prior to hospitalisation, n (%) | 3 (20)  7 (47) | 6 (38)  2 (13) | 2 (11)  5 (28) | | NS  NS |
| Multifocal infection, n (%)  Bacteraemia, n (%)  Methicillin-resistant Staphylococcus aureus | 10 (67)  11 (73)  9 (60) | 12 (75)  11 (69)  9 (56) | | 9 (50)  14 (78)  12 (67) | NS  NS  NS |
| SAPS II score, median [IQR] | 50 [25-81] | 29 [15-51] | | 41 [31-56] | NS |
| Mechanical ventilation  Vasoactive support, n (%)  Surgical procedure for source control, n (%)  Adequate empiric antibiotic therapy, n (%)  Adequate documented antibiotic therapy, n (%)  Intravenous immunoglobulin therapy, n (%) | 11 (73)  11 (73)  6 (40)  8 (53)  14 (93)  11 (73) | 11 (69)  6 (38)  11 (69)  11 (69)  16 (100)  12 (75) | | 9 (50)  9(50)  8 (44)  11 (61)  17 (94)  14 (78) | NS  NS  NS  NS  NS  NS |
| Main complications  Adult respiratory distress syndrome, n (%)  Multiple organ failure, n (%)  Duration of ICU stay of survivors, median days [IQR]  Death, n (%) | 5 (36)  8 (53)  10 [3-26]  6 (40) | 5 (31)  4 (25)  5 [2-27]  2 (13) | | 8 (44)  5 (28)  10 [5-28]  2 (11) | NS  NS  NS  NS |
